# Optical Genome Mapping for the Molecular Diagnosis of Facioscapulohumeral Muscular Dystrophy: Advancement and Challenges

**DOI:** 10.3390/biom13111567

**Published:** 2023-10-24

**Authors:** Stephanie Efthymiou, Richard J. L. F. Lemmers, Venugopalan Y. Vishnu, Natalia Dominik, Benedetta Perrone, Stefano Facchini, Elisa Vegezzi, Sabrina Ravaglia, Lindsay Wilson, Patrick J. van der Vliet, Rinkle Mishra, Alisha Reyaz, Tanveer Ahmad, Rohit Bhatia, James M. Polke, Mv Padma Srivastava, Andrea Cortese, Henry Houlden, Silvère M. van der Maarel, Michael G. Hanna, Enrico Bugiardini

**Affiliations:** 1 Department of Neuromuscular Disorders, UCL Queen Square Institute of Neurology, London WC1N 3BG, UK; s.efthymiou@ucl.ac.uk (S.E.); n.dominik@ucl.ac.uk (N.D.); b.perrone@ucl.ac.uk (B.P.); lindsay.wilson@ucl.ac.uk (L.W.); h.houlden@ucl.ac.uk (H.H.);; 2 Department of Human Genetics, Leiden University Medical Center, 2333 ZA Leiden, The Netherlandss.m.van_der_maarel@lumc.nl (S.M.v.d.M.); 3 Department of Neurology, All India Institute of Medical Sciences, New Delhi 110029, India; vishnuvy16@yahoo.com (V.Y.V.); rinklemishra1994@gmail.com (R.M.); alisha03r@gmail.com (A.R.); rohitbhatia71@yahoo.com (R.B.); vasanthapadma123@gmail.com (M.P.S.); 4 IRCCS Mondino Foundation, 27100 Pavia, Italy; elisa.vegezzi@mondino.it (E.V.); sabrina.ravaglia@mondino.it (S.R.); 5 Neurogenetics Laboratory, National Hospital for Neurology and Neurosurgery, London WC1N 3BG, UK; james.polke@nhs.net

**Keywords:** FSHD, D4Z4 contraction, optical genome mapping, Bionano Genomics

## Abstract

Facioscapulohumeral muscular dystrophy (FSHD) is the second most common muscular dystrophy in adults, and it is associated with local D4Z4 chromatin relaxation, mostly via the contraction of the D4Z4 macrosatellite repeat array on chromosome 4q35. In this study, we aimed to investigate the use of Optical Genome Mapping (OGM) as a diagnostic tool for testing FSHD cases from the UK and India and to compare OGM performance with that of traditional techniques such as linear gel (LGE) and Pulsed-field gel electrophoresis (PFGE) Southern blotting (SB). A total of 6 confirmed and 19 suspected FSHD samples were processed with LGE and PFGE, respectively. The same samples were run using a Saphyr Genome-Imaging Instrument (1-color), and the data were analysed using custom EnFocus FSHD analysis. OGM was able to confirm the diagnosis of FSHD1 in all FSHD1 cases positive for SB (*n* = 17), and D4Z4 sizing highly correlated with PFGE-SB (*p* < 0.001). OGM correctly identified cases with mosaicism for the repeat array contraction (*n* = 2) and with a duplication of the D4Z4 repeat array. OGM is a promising new technology able to unravel structural variants in the genome and seems to be a valid tool for diagnosing FSHD1.

## 1. Introduction

Facioscapulohumeral muscular dystrophy (FSHD) is the second most common muscular dystrophy in adults, with a prevalence of nearly 1:8000 [1]. It causes muscle weakness initially affecting the muscles in the face and around the scapula and then progressively affecting the arm and legs. Up to 20% of affected individuals can become wheelchair-dependent by the age of 50. About 95% of FSHD cases are associated with a contraction of the D4Z4 macrosatellite repeat array in the subtelomeric region of chromosome 4q35 [2,3], and these cases are defined as FSHD type 1. In the European population, healthy individuals have between 8 and 100 D4Z4 repeats, whereas people affected by FSHD have 1 to 10 repeats. Two main haplotypes have been described for 4q35, namely,4qA and 4qB, with 4qA alleles being permissive for FSHD, whereas D4Z4 repeat array contractions on 4qB chromosomes are not associated with disease [4].

An FSHD diagnosis is not achievable using the short-read next-generation-sequencing (NGS) techniques now widely used for the diagnosis of genetic diseases. In most laboratories, FSHD1 diagnosis is based on the Southern blot (SB) method [4,5]. In this method, genomic DNA is digested by the restriction enzyme EcoRI, and the D4Z4 fragments from chromosomes 4 and 10 are visualised after separation via gel electrophoresis, blotting, and hybridisation with probe p13E-11. Discrimination between chromosomes 4 and 10 is achieved using double digestion with EcoRI and BlnI or via the restriction enzyme XapI, where chromosome 4 repeat repeats are resistant to BlnI and sensitive to XapI, and vice versa for chromosome-10-derived repeats. Discrimination between 4qA and 4qB occurs on a separate blot, where DNA is digested with HindIII and hybridized with probes A and B. However, there are complex genetic situations that can lead to false positive or false negative results [4,6] that can complicate SB interpretation, such as (1) FSHD2, a rare digenic condition caused by a combination of an intermediate D4Z4 repeat array with a size of 8–20 units on an 4qA chromosome and a mutation in a D4Z4 chromatin modifier [7]; (2) the presence of translocations between chromosomes 4 and 10 [5]; (3) somatic mosaicism, where there are two cell populations, one with a de novo contracted repeat array and one with a parental-sized repeat array—consequently, this situation reveals three chromosome 4 alleles for which the mosaic alleles show a lower signal intensity [8]; (4) the presence of non-canonical deletions, including of the region recognised by the p13E-11 probe [9]; and (5) duplications of the D4Z4 repeat array [10,11]. FSHD molecular diagnosis based on SB is often also challenged by technical difficulties because of the quality of biological samples and SB blots. Current guidelines on the genetic diagnosis of FSHD suggest a stepwise approach with an increasing number of tests performed depending on the initial results and whether the test was requested for confirming FSHD (typical FSHD) or excluding FSHD (atypical FSHD) [12,13]. Therefore, SB protocols in relation to FSHD are technically challenging, time consuming, and require staff trained in results interpretation. 

A growing number of technologies are currently being analysed in an attempt to overcome the challenges regarding SB technology, including molecular combing, a technology with which the D4Z4 repeat arrays are visualized via fluorescent in situ hybridisation on stretched DNA molecules [11,14]; optical genome mapping (OGM) [15]; and long-read sequencing-based approaches [16]. OGM can be used to read single, unamplified, long fragments of DNA with an average length of 300 kb, thus mitigating the bias of large structural and copy-number variation. Currently, the cost of OGM is greater than that of SB (on the order of three times higher). However, the majority of the excess cost comes from consumables, while the processing is shorter and requires less technical skill. Reagent costs are expected to drop in the next few years as demand grows, while SB’s cost is not expected to change. 

Currently, only a few laboratories worldwide offer full FSHD genotyping, and access to the FSHD genetic diagnosis of populations in low- and middle-income countries (LMIC) such as India is limited, with negative implications for genetic and clinical counselling. Because a greater number of clinical trials are now available for FSHD and it is possible that a disease-modifying treatment will be developed soon, there is an urgent need to provide access to FSHD genetic diagnosis worldwide.

Here, we present a performance comparison between OGM and traditional SB analysis for the molecular analysis and characterisation of FSHD subtypes. We evaluate the accuracy and reproducibility of OGM in assessing the D4Z4 repeat array allele sizes and A and B haplotype sequences necessary to confirm the diagnosis of FSHD1 or FSHD2. Additionally, we test the ability of OGM to assess complex cases such as mosaicism and duplications. We visually evaluate complete OGM data to determine their efficacy in confident mapping proximally to either chromosome by virtue of the length and density of the fluorescent tags on the individual molecules proximal to the D4Z4 repeat array.

## 2. Materials and Methods

### 2.1. Subjects

This study was approved by the Institutional Review Boards of UCL Hospital (UCLH) and the All India Institute of Medical Sciences (AIIMS) New Delhi (see ethics), and OGM testing was performed in the Neurogenetics Laboratory at the UCL Queen Square Institute of Neurology. We recruited 6 patients from the UK with genetically confirmed FSHD1 and 1 with a case of confirmed FSHD2. Three additional UK cases were included with clinically confirmed FSHD but with negative diagnostic FSHD test results as determined via linear gel electrophoresis (LGE) SB. All cases from the UK attended clinics at the National Hospital for Neurology and Neurosurgery (NHNN). A total of 15 cases with suspected FSHD from India (Gujarati region, North India) were tested using both pulsed-field gel electrophoresis (PFGE) and OGM. These cases belong to a broader Indian FSHD cohort currently under investigation. A subset (*n* = 4) of these samples was initially tested using SB and selected for this study due to the presence of complex genetic rearrangements (cis-duplication or mosaicism).

### 2.2. SB Analysis

Each UK specimen had previously undergone diagnostic genetic testing using restriction enzyme digests with either EcoRI, EcoRI/BlnI, and XapI followed by LGE SB and p13E-11 hybridisation (conducted at North Bristol NHS Trust). PFGE and SB analyses were conducted using the restriction enzymes EcoRI/HindIII, EcoRI/BlnI, XapI (for repeat sizing), or HindIII (for haplotyping), followed by hybridisation with appropriate probes. D4Z4 methylation analysis was performed on the cohort from India using the methylation-sensitive restriction enzyme FseI, and methylation levels were corrected for repeat sizes (delta values) (conducted at the department of Human Genetics at Leiden University Medical Center) [4,7].

### 2.3. Sample Preparation and OGM

Fresh blood samples from UK-based patients were aliquoted on the day of collection into 450 µL blood aliquots and stored at 80 °C. For Indian-based patients, fresh blood was received at the UCL lab within 7 days of collection, after which aliquots were made and frozen at −80 °C. 

We used OGM technology (Bionano Genomics, San Diego, CA, USA) to decipher the genomic architecture of our region of interest, specifically to determine size and haplotype of D4Z4 alleles. High-molecular-weight DNA was isolated from fresh blood using the Specimen Preparation Fresh Human Blood DNA Isolation Protocol (Bionano Genomics; catalogue #80042). Occasionally, archived agarose plugs were used as an alternative to blood samples, and high-molecular-weight DNA was isolated from fresh blood using the Prep Blood and Cell Culture DNA Isolation Kit (Bionano Genomics, catalogue #80004). Genomic DNA was fluorescently tagged with Direct Labeling Enzyme 1 by using the Direct Label and Stain DLS DNA Kit (Bionano Genomics, catalogue #80005). Labelled DNA molecules were electrophoresed through low-voltage nanochannel arrays on a Saphyr chip v2.2 (Bionano Genomics; catalogue #20366) to linearize the DNA. 

High-throughput sequential imaging of the nanochannels was performed by using a Saphyr Genome Imaging Instrument (Bionano Genomics, San Diego, CA, USA) to produce thousands of high-resolution images from which the molecule maps could be derived. The throughput target for each sample was 500 Gbp for an expected effective coverage of at least 80×; data collection typically took around 12 h. Data were processed using Bionano Solve software version ICS 5.2.21307.1 to align labelled molecules against the reference sequence-predicted label pattern; the hg38 reference carries both the 4qA and 4qB D4Z4 haplotypes [4]. Molecules aligned to the reference chromosome 4q35 or 10q26 regions were further collected to generate representative allelic profiles of structural variation to interpret FSHD genotypes using a custom EnFocus FSHD analysis (version 1.0; Bionano Genomics). Samples with insufficient data were further analysed using the de novo assembly for full genomes. 

All chromosome 4 and 10 repeat array sizes and haplotypes identified via OGM were compared with SB analysis data based on LGE and on PFGE.

## 3. Results

### 3.1. Data Quality and Processing

We evaluated the sensitivity of OGM with respect to characterizing the D4Z4 repeat arrays in a total of 25 samples with known or suspected FSHD. The OGM performed on the genomic DNA of each specimen resulted in minimal deviation in quality control metrics and produced highly interpretable results for D4Z4 allele size and haplotype (Table 1). The map rate (the percentage of molecules ≥150 kbp that aligned with the reference) ranged from 42% to 95%; the effective coverage was, as a mean, 118×, with 92% samples above 70×; and the DNA length was stable, with N50 measurements ranging from 185 to 382 Kbp.

### 3.2. OGM vs. LGE

To assess OGM performance against the UK standard diagnostic test (LGE), we processed six FSHD1 genetically confirmed cases, all of which were correctly identified via OGM. We also processed an FSHD2 case (NHNN_FSH005) with an *SMCHD1* (c.3927 + 1G > C) pathogenic variant and a known average DR1 hypomethylation (19%), and OGM was able to size the intermediate-sized D4Z4 fragment (18 U), which was not detectable via standard LGE. We then processed three cases attending our clinic at NHNN with a high clinical suspicion of FSHD and previous negative diagnostic FSHD test results. One case was found with homozygous 10 U on 4qA, and a second case (NHNN-010) presented a D4Z4 cis-duplication. The third case resulted negative.

### 3.3. OGM vs. PFGE

We then wanted to explore whether OGM is a technology that can be considered for FSHD diagnostics in India. All the analysed cases had suspected FSHD but had never been formally tested because genetic testing for FSHD was not available in India. Therefore, the samples were tested in parallel with standard FSHD diagnostics (PFGE SB). Shipping at room temperature for up to 7 days did not affect the results (Table 1) for the 15 cases tested. 

**Figure 1:**
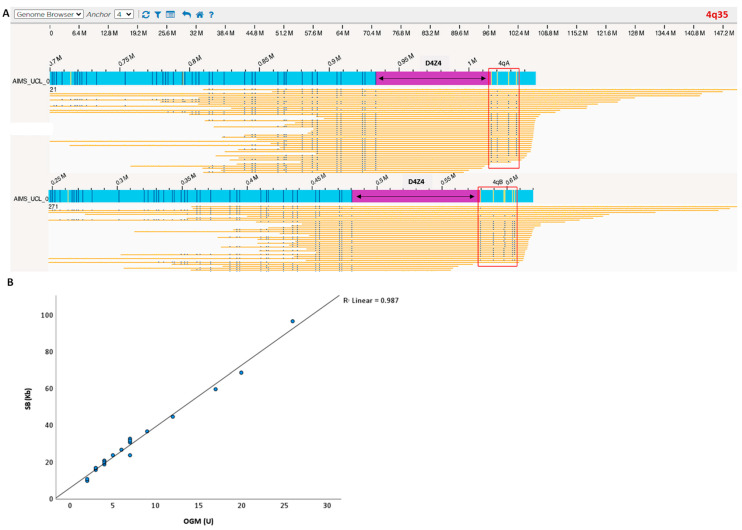
(**A**) Case example processed with OGM. Blue bar is the sample consensus map; yellow bars are individual sample molecules; D4Z4 region is highlighted in purple. The blue vertical lines represent the positions of the fluorescent labels (OGM markers). The number of D4Z4 repeats was calculated by measuring the distance between the most proximal and distal OGM markers. Label patterns are also used to differentiate the haplotypes A and B (red square). (**B**) OGM detected a 4q35 D4Z4 macrosatellite contraction ranging from 1 to 26 D4Z4 repeats units and showed a strong correlation with SB allele sizing and haplotyping (100% haplotype match). Blue dots represent individual patients D4Z4 sizing. Linear regression showed that repeat array allele size according to OGM correlates well with the size determined via SB analysis.

Nine individuals were diagnosed with FSHD1, and two were determined to have FSHD2 due to the presence of severe hypomethylation (Table 1). In one of them, IC_AIM_000932 (9 U), a pathogenic variant in *SMCHD1*, was identified (c.3051_3075del, p.Ser1017Arg*fs*Ter20). For the remaining cases, two carried a cis duplication allele that is probably pathogenic, and one turned out to be negative (D4Z4 20 U). OGM was able to confirm all FSHD cases, and D4Z4 sizing highly correlated with the data from SB (*p* < 0.001) (Figure 1B). Furthermore, haplotype assignments were consistent with the SB data in all the cases analysed.

### 3.4. Complex FSHD Cases

During the study, OGM highlighted interesting cases that were missed when using standard LGE. The three main categories analysed are as follows. 

#### 3.4.1. Somatic Mosaicism

**Figure 2:**
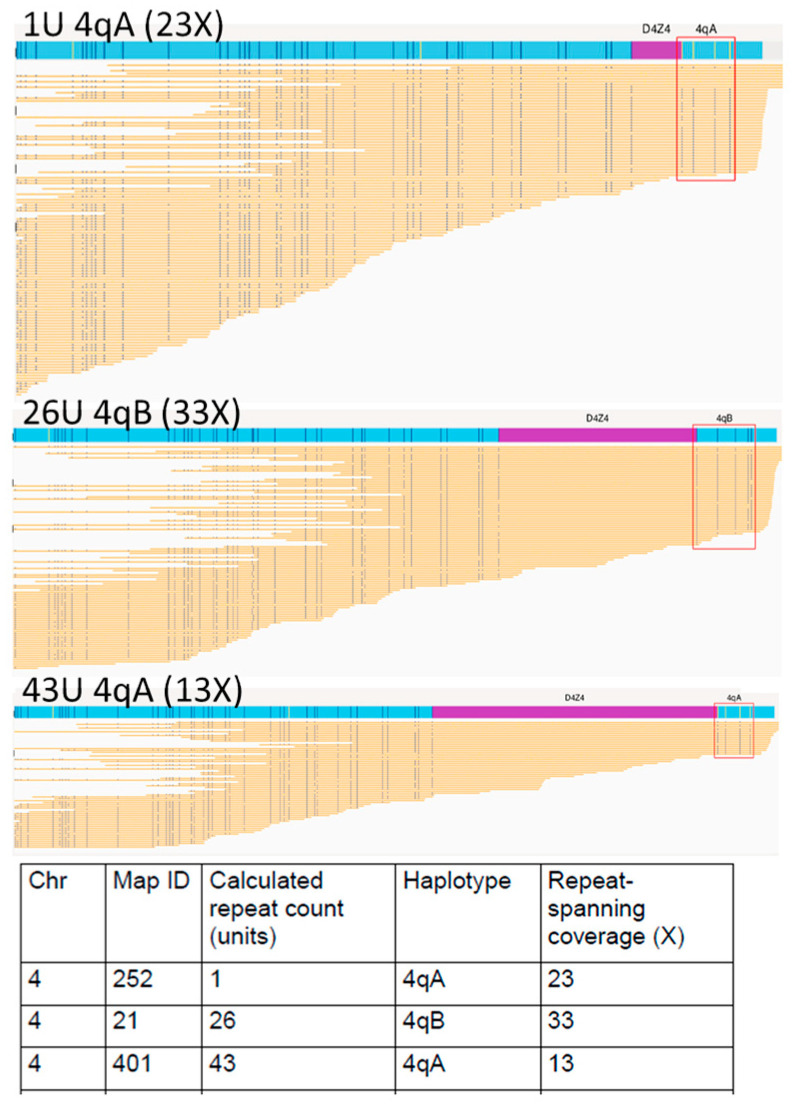
Optical genome mapping (EnFocus FSHD analysis (version 1.0; Bionano Access Software v1.7) for the analysis of proband NHNN_FSH006 with somatic mosaicism. The output files show all reads aligned to the two D4Z4 alleles on chromosome 4. Specific fluorescence tags proximal and distal to the D4Z4 arrays enabled chromosomal assignment and A-B haplotyping. The fluorescence tags are absent in the D4Z4 region; here, the number of D4Z4 units was derived from the size of this unlabelled region.

OGM and PFGE were able to accurately detect somatic mosaicism on chromosome 4 in two cases. One of them (UK case NHNN_FSH006) was identified as non-mosaic via LGE, presumably because the proportion of the mosaic FSHD1 allele was too high and because the other D4Z4 alleles (including the normal-sized parental mosaic allele) were not shown (Figure 2) (see reference about LGE and detection of mosaicism [8]). The identification of mosaic FSHD1 fits with the observed clinical severity, which was lower than that usually observed for non-mosaic carriers of a 1 U FSHD allele.

#### 3.4.2. Homozygous

**Figure 3:**
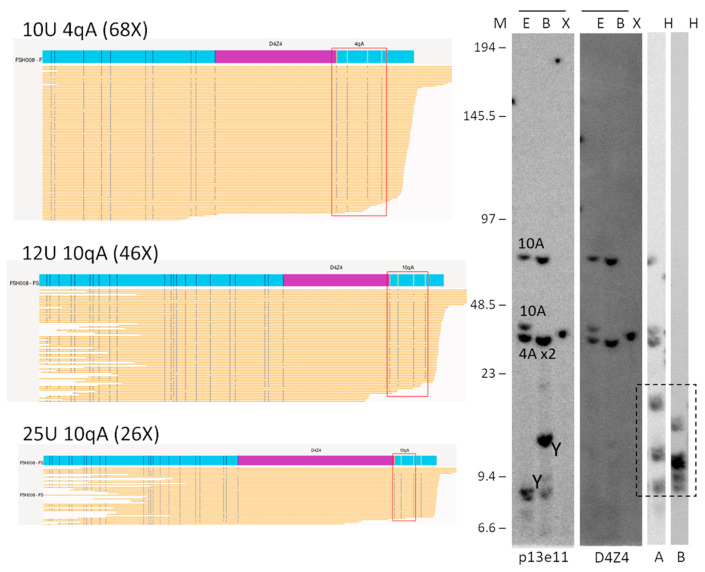
Comparison of OGM (EnFocus FSHD analysis (version 1.0; Bionano Access Software v1.7)) and SB hybridisation and SSLP haplotyping for the analysis of proband FSH008 with homozygous 10 U alleles. The OGM output files (left panel) show one Chr 4 map with a higher number of reads, suggesting the presence of a homozygous D4Z4 region. SB analysis (**right** panel) showed hybridisations with probes p13E-11 and D4Z4 on genomic DNA digested using the enzymes EcoRI/HindIII €, EcoRI/BlnI (B), and XapI (X) and hybridisations with probes A and B on genomic DNA digested with HindIII (H). The alleles based on the different blots are indicated in the p13E-11 blot. The chromosome Y fragment is indicated, and the cross-hybridising fragments in the A/B hybridisations are marked with a dotted box. The size of the molecular weight marker is indicated on the **left**. Original blot images can be found in Figure S1.

One patient presenting clinically as having FSHD with facial, shoulder, and pelvic girdle weakness was tested via LGE and considered borderline-negative (size ~44 Kb). OGM was able to accurately measure the contraction size on the 4qA allele, which was 10 U, and, based on the number of reads and the lack of a second allele, suggested the presence of two chromosome 4 alleles with a size of 10 U. the results were then confirmed via PFGE (Figure 3). The presence of two copies with a contracted allele of 10 U suggests a potential causative role of the D4Z4 contraction in the patient’s clinical manifestation. 

#### 3.4.3. Duplication

**Figure 4:**
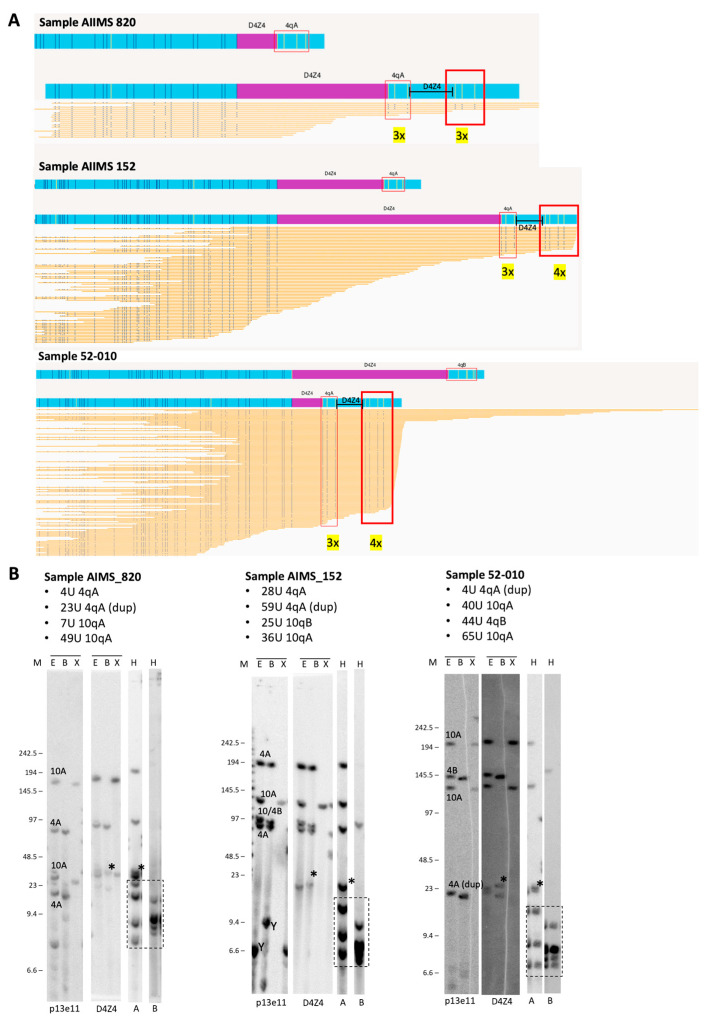
(**A**) Duplicated 4qA signatures identified via visual inspection of OGM data using EnFocus FSHD analysis browser view (version 1.0; Bionano Access Software v1.7) for the analysis of probands AIIMS 820, AIIMS 152, and 52-010. The OGM output files show that all reads aligned to the D4Z4 alleles on chromosome 4. Nick sites are denoted by the red box, and the number of sites is given in yellow. (**B**) Comparison with SB analysis shows hybridisations with probes p13E-11 and D4Z4 on genomic DNA digested using the enzymes EcoRI/HindIII (E), EcoRI/BlnI (B), and XapI (X) and hybridisations with probes A and B on genomic DNA digested with HindIII (H). The alleles based on the different blots are indicated in the p13E-11 blot. The chromosome Y fragment is indicated, and the cross-hybridising fragments in the A/B hybridisations are marked with a dotted box. The size of the molecular weight marker is indicated on the left, and the asterisk (*) indicates the extra allele that appears upon D4Z4 and 4qA hybridisation and depicts the duplication allele. Original blot images can be found in Figure S1.

Two cases from the India cohort were identified as having a D4Z4 cis-duplication via PFGE-based SB (AIMS_820 and AIMS_152). Interestingly, the currently available EnFocus analysis was not able to directly detect and determine the duplication in the generated report. However, when the EnFocus browser view was visually inspected, duplicated signatures were identified in all cases [18]. When we re-assessed via visual inspection the UK FSHD cases with negative genetics, we identified a third case (NHNN-FSH010) with a similar signature (Figure 4) suggesting the presence of a duplication. This case previously tested negative for FSHD2 (with a DR1 BS-PCR methylation level of 39.1% and negative SMCHD1 gene-sequencing results).

## 4. Discussion

FSHD diagnostics in accredited laboratories mostly rely on SB analysis either following LGE or sometimes PFGE. In the UK, LGE is available, and haplotype analysis is only performed in cases of unclear results after initial testing. OGM is emerging as a promising technology for FSHD diagnostics and a suitable alternative to SB as it enables repeat sizing and haplotyping in one go [19,20]. 

In this study, we assessed the use of OGM as a diagnostic tool for assessing FSHD, and we compared its performance to that of traditional diagnostic techniques, such as SB, with a specific focus on complex FSHD cases. We confirmed that OGM is a sensitive and accurate tool for FSHD diagnosis. All positive cases determined via SB (either PFGE or LGE) were also positive, with OGM and D4Z4 repeat size and haplotype assignment showing high concordance. This is in line with recent data on OGM [20]. 

Of note, we also showed the feasibility of testing samples shipped from abroad. The current recommendation from the manufacturers is to process blood within 4 days of the extraction date and without additional freeze/thaw cycles. In this study, OGM was performed on specimens either delivered on the same day from the NHNN clinic or posted over 4–5 days from India. Once aliquoted and frozen at −80 °C, the 500 µL aliquoted specimens were thawed and processed for DNA isolation within a month. Importantly, we confirmed that using fresh blood from Indian-based patients within 7 days of extraction transported at room temperature is sufficient for isolating high-molecular-weight DNA that can be labelled. Finally, we tested a few samples using plug DNA extracted previously, obtaining high-quality OGM results, suggesting that this can be an alternative method for testing samples in settings where fresh samples shipped from long distances can pose challenges.

In our study, we also showed that the performance of OGM was superior to LGE SB and equal to that of PGFE SB for the cases analysed. Indeed, OGM and PFGE showed excellent correlations in repeat sizing and haplotyping. LGE-SB, however, has the intrinsic limitation that it cannot be used to provide a full analysis of the four D4Z4 alleles from chromosomes 4 and 10, with the risk of incomplete diagnosis and increasing the number of false negatives cases. This has important implications for genetic counselling and for access to the ongoing clinical trials and, hopefully, treatment in the near future. In our study, only a small, carefully selected cohort of FHSD-like cases was chosen, presenting very high clinical suspicion and clinical features in keeping with FSHD but determined to be negative for this disease via LGE. In this cohort, two cases out of three that were reported to be negative via LGE showed a complex genetic background when analysed using OGM. One sample (NHNN_FSH008) had a borderline negative result as determined via SB (size ~44 Kb). First, OGM showed a more precise sizing of 10 U, in keeping with FSHD; second, it highlighted a homozygous 10 U zone on Chr4 undetectable via LGE SB. There is a debate as to whether people with compound heterozygous or homozygous variants could have a more severe phenotype [21,22]. More data are needed to establish this, but certainly identifying homozygous cases not detectable via LGE has important implications for genetic counselling. The other case was identified as having a cis D4Z4 repeat array duplication in FSHD. Previously, cis duplication alleles were suggested to only be pathogenic in combination with an SMCHD1 mutation in FSHD2, but more recently, they have been identified as being dominantly pathogenic for FSHD in certain conditions [11,18]. Of note, this case was completely missed using LGE. Another benefit of OGM over LGE was the identification of mosaic cases (NHNN_FSH006). Having these data available is important in order to determine clinical and genetic implications, and the current diagnostic strategy in the UK with regard to LGE is limited in providing this information. One limitation of OGM is that it does not allow for simultaneous methylation analysis, which is currently needed to support a diagnosis of FSHD2.

Finally, in our study, we demonstrated that OGM can be used to detect cis D4Z4 repeat array duplications, albeit only after manual inspection. Cis D4Z4 duplications are increasingly being recognised as a potential cause of FSHD [23] and can easily be missed by diagnostic laboratories. The Standard Bionano EnFocus pipeline cannot detect these duplications and report them as part of the generated report. However, we were able to detect them via visual inspection, in which they appeared as a duplication of the haplotype signature. Our data are supported by a recent publication on complex rearrangement, including cis duplications, assessed via molecular combining and OGM [18] that used a similar approach to identify cis duplications. Interestingly, four nick sites exist immediately distal to D4Z4 that can be used to define the Haplotype A, but in some D4Z4 repeat array duplications, only that sites are visible. This may be an artefact, as the DNA fragment containing the cis duplication might be not long enough to cover the full four nick sites, and it also implies that the successful detection of cis duplications via OGM relies on the presence of nick sites in the spacer sequence in between the duplicated D4Z4 repeat arrays.

In conclusion, OGM can be used to deliver a diagnosis for people with FSHD, including complex cases missed when using LGE SB. The relative simplicity of this protocol and its ability to identify all repeat array sizes and haplotypes in one go makes it an accessible tool for many genetic laboratories worldwide, allowing genetic testing for FSHD globally. Further health economics evaluation will be needed to understand if OGM can deliver improved access to FSHD diagnostic testing in low- and middle-income settings.

## Figures and Tables

**Table 1:** List of participants included in this study. Comparison of pathogenic 4q35A allele characteristics using SB and OGM as well as details on the specimen stability used in OGM. Optical genome mapping was performed on specimens stored and processed under two representative conditions: 7 days (d) post-collection and subsequent extraction for Indian samples, and 1 day (d) post-collection and subsequent extraction for UK samples. Four additional samples were tested using archived agarose plugs. SB results were compared with those of OGM. N50 (>0.2) is used as a surrogate marker of molecule size; N50 values represent weighted average lengths of DNA molecules that are 20 Kbp or longer. Map rate (%) indicates the percentage of molecules 150 Kbp or longer that map to the reference genome. Effective coverage indicates the total amount of aligned molecules divided by the length of the reference genome multiplied by the map rate. SB-LGE: Southern blot and Linear Gel Electrophoresis; SB-PFGE: Southern blot and Pulsed-Field Gel Electrophoresis; NA; not analysed; ND: not detected, assumed to be >48 Kb; Methylation FSHD2 thresholds: delta1 ≤ 20%.

Specimen ID ^#^	Source	Methylation	SB-LGE		SB-PFGE					OGM					Shipping/Storing	Molecules ≥ 150 Kbp	N50 > 0.2	Map Rate (%)	Effective Coverage
			4q_1	Outcome	4q_1		4q_2		Outcome	4q_1		4q_2		Outcome					
		FseI (delta1) *	U		U	A/B	U	A/B		U	A/B	U	A/B						
NHNN_FSH001	UK	NA	>10	No F1 No F2	23	B	24	B	Negative	23	B	24	B	No F1, No F2	d1, −80 °C	332	0.277	94	166×
NHNN_FSH002	UK	NA	7	FSHD1	NA	NA	NA	NA	-	7	A	49	A	FSHD1	d1, −80 °C	304	0.248	95	151×
NHNN_FSH003	UK	NA	6	FSHD1	NA	NA	NA	NA	-	6	A	39	A	FSHD1	d1, −80 °C	382	0.340	93	147×
NHNN_FSH004	UK	NA	8	FSHD1	NA	NA	NA	NA	-	7	A	29	A	FSHD1	d1, −80 °C	242	0.186	91	142×
NHNN_FSH005	UK	NA	>10	FSHD2	NA	NA	NA	NA	-	18	A	24	B	FSHD2	d1, −80 °C	293	0.228	65	106×
NHNN_FSH006	UK	NA	1	FSHD1 ^#^	NA	NA	NA	NA	-	[1(64%);43(36%)]	A	26	B	FSHD1 mosaic	d1, −80 °C	258	0.198	91	172×
NHNN_FSH007	UK	NA	5	FSHD1	NA	NA	NA	NA	-	5	A	27	B	FSHD1	d1, −80 °C	220	0.158	88	145×
NHNN_FSH008	UK	−13	11	No F1, No F2	10	A	10	A	FSHD1 ^^^	10	A	10	A	FSHD1 ^^^	d1, −80 °C	213	0.143	79	122×
NHNN_FSH009	UK	NA	8	FSHD1	NA	NA	NA	NA	-	7	A	18	A	FSHD1	d1, −80 °C	203	0.120	75	83×
NHNN_FSH010	UK	NA	>10	No F1, No F2	13 + 6	A	22	A	FSHD dup	13 + D	A	22	A	FSHD dup	d1, −80 °C	203	0.142	83	131×
IC_AIM_000950	India	0	NA	-	12	B	43	A	No F1, No F2	12	B	42	A	No F1, No F2	4 °C, d7, −80 °C	251	0.131	80	134×
IC_AIM_000918	India	NA	NA	-	2	A	23	B	FSHD1	2	A	23	B	FSHD1	4 °C, d7, −80 °C	237	0.126	81	136×
IC_AIM_000955	India	NA	NA	-	5	A	22	A	FSHD1	4	A	22	A	FSHD1	4 °C, d7, −80 °C	228	0.118	74	105×
IC_AIM_000957	India	NA	NA	-	5	A	39	A	FSHD1	4	A	38	A	FSHD1	4 °C, d7, −80 °C	249	0.103	77	123×
IC_AIM_000904	India	NA	NA	-	3	A	24	B	FSHD1	3	A	24	B	FSHD1	4 °C, d7, −80 °C	220	0.094	67	107×
IC_AIM_000963	India	NA	NA	-	4	A	25	A	FSHD1	4	A	25	A	FSHD1	4 °C, d7, −80 °C	270	0.158	83	132×
IC_AIM_000932	India	−35	NA	-	9	A	12	B	FSHD2	9	A	12	B	FSHD2	4 °C, d7, −80 °C	236	0.105	80	122×
IC_AIM_001042	India	−41	NA	-	19	A	19	A	FSHD2	20	A	20	A	FSHD2	4 °C, d7, −80 °C	221	0.090	38	62×
IC_AIM_001045	India	NA	NA	-	5	A	25	B	FSHD1	4	A	25	B	FSHD1	4 °C, d7, −80 °C	213	0.100	64	112×
IC_AIM_001053	India	NA	NA	-	4	A	21	B	FSHD1	3	A	21	B	FSHD1	4 °C, d7, −80 °C	235	0.147	78	123×
IC_AIM_001055	India	NA	NA	-	6	A	25	A	FSHD1	7	A	>11	ND	FSHD1	4 °C, d7, −80 °C	185	0.083	42	66×
IC_AIM_000820	India	NA	NA	-	4	A	23 + 6	AD	FSHD1	3	A	24 + D	A	FSHD1	4 °C, d5	212	0.161	78	51×
IC_AIM_00152	India	16	NA	-	28	A	59 + 3	AD	FSHD dup	26	A	61 + D	A	FSHD dup	4 °C, d5	289	0.234	88	102×
IC_AIM_000701	India	NA	NA	-	[2(40%);20(60%)]	A	10	B	FSHD1 mosaic	[2(36%);21(64%)]	A	10	B	FSHD1 mosaic	4 °C, d5	260	0.177	80	128×
IC_AIM_000531	India	−8	NA	-	17	B	20	A	No F1, No F2	17	B	21	A	No F1, No F2	4 °C, d5	250	0.167	74	89×

* delta1 values represent repeat size-corrected D4Z4 methylation calculated as described in Lemmers et al. 2014 [17]. ^#^ mosaicism character of the FSHD1 allele was not identified by SB-LGE in sample NHNN_FSH006. ^^^ homozygous 10 U. NHNN_FSHD008 has a phenotype consistent with FSHD (facial weakness; shoulder and pelvic girdle weakness) and was found to contain Homozygous 10 U via OGM.

## Data Availability

The data presented in this study are available on request from the corresponding author.

## Supplementary Materials

The following supporting information can be downloaded at: https://www.mdpi.com/article/10.3390/biom13111567/s1, Figure S1: original blot images.
