# Supplementary figures and images for: Optical Genome Mapping for the Molecular Diagnosis of Facioscapulohumeral Muscular Dystrophy: Advancement and Challenges
Source: Biomolecules. 2023 Oct 24;13(11):1567. doi: 10.3390/biom13111567 (PMC10669274; doi:10.3390/biom13111567)

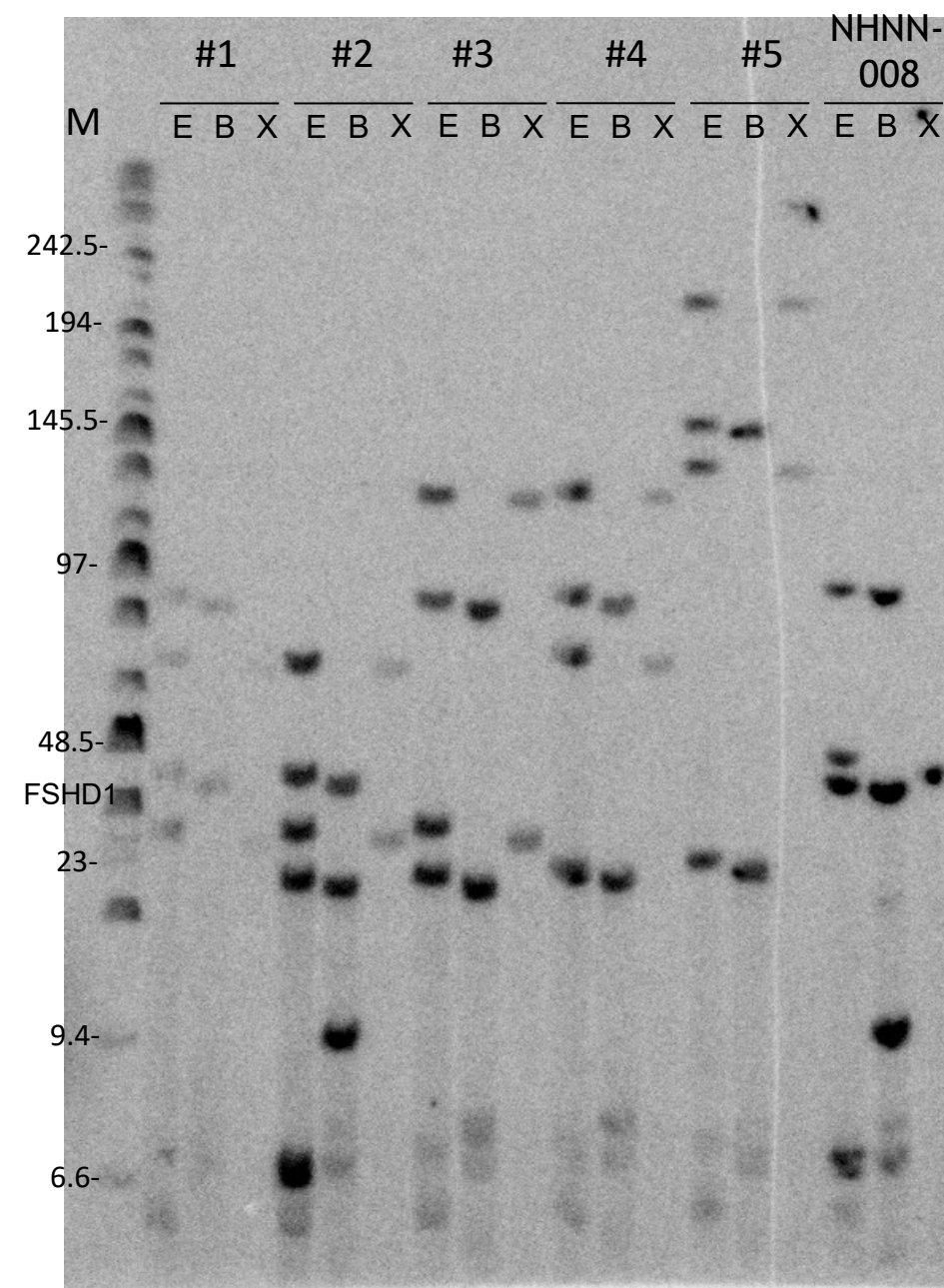

1) p13E-11

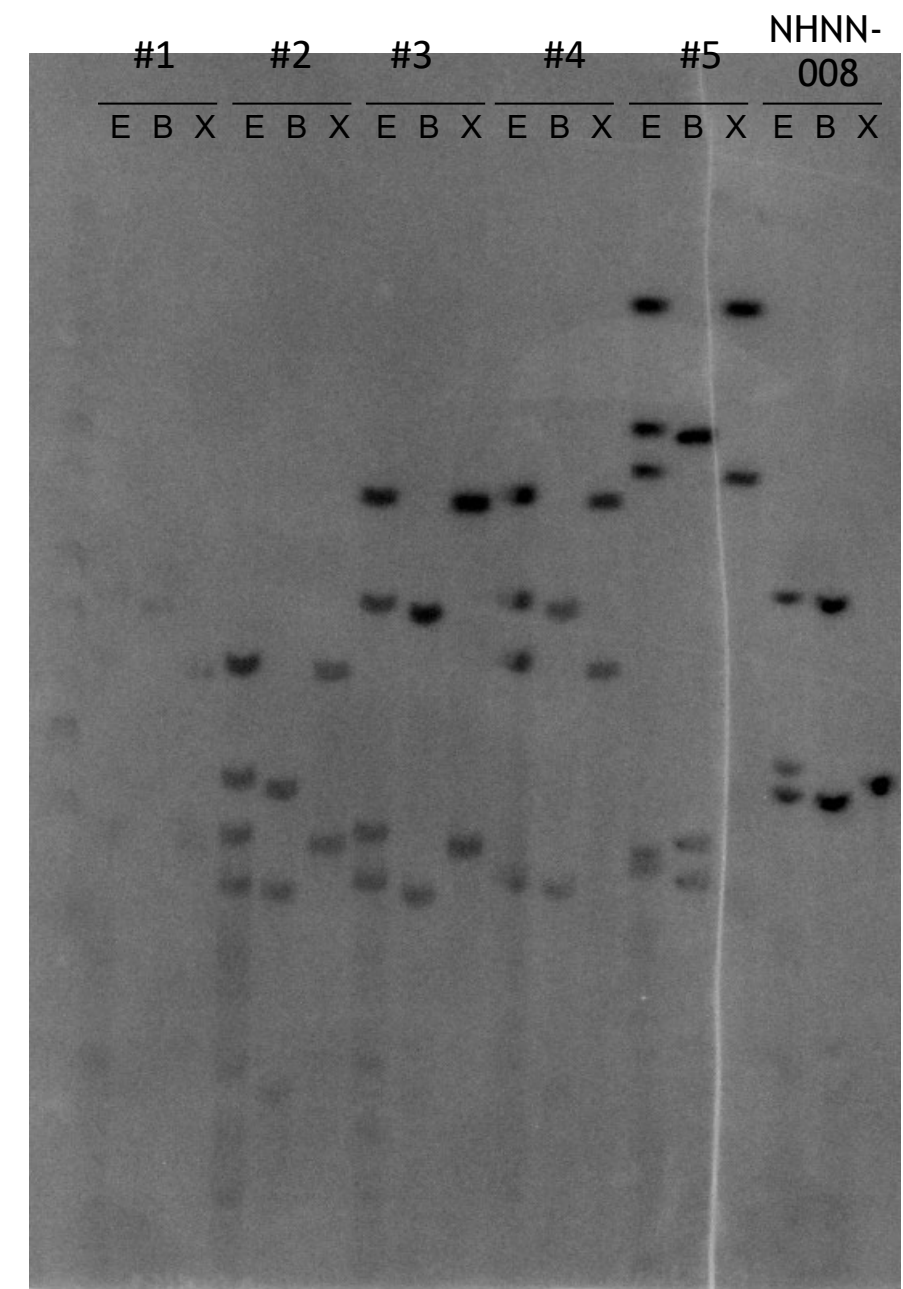

2) D4Z4

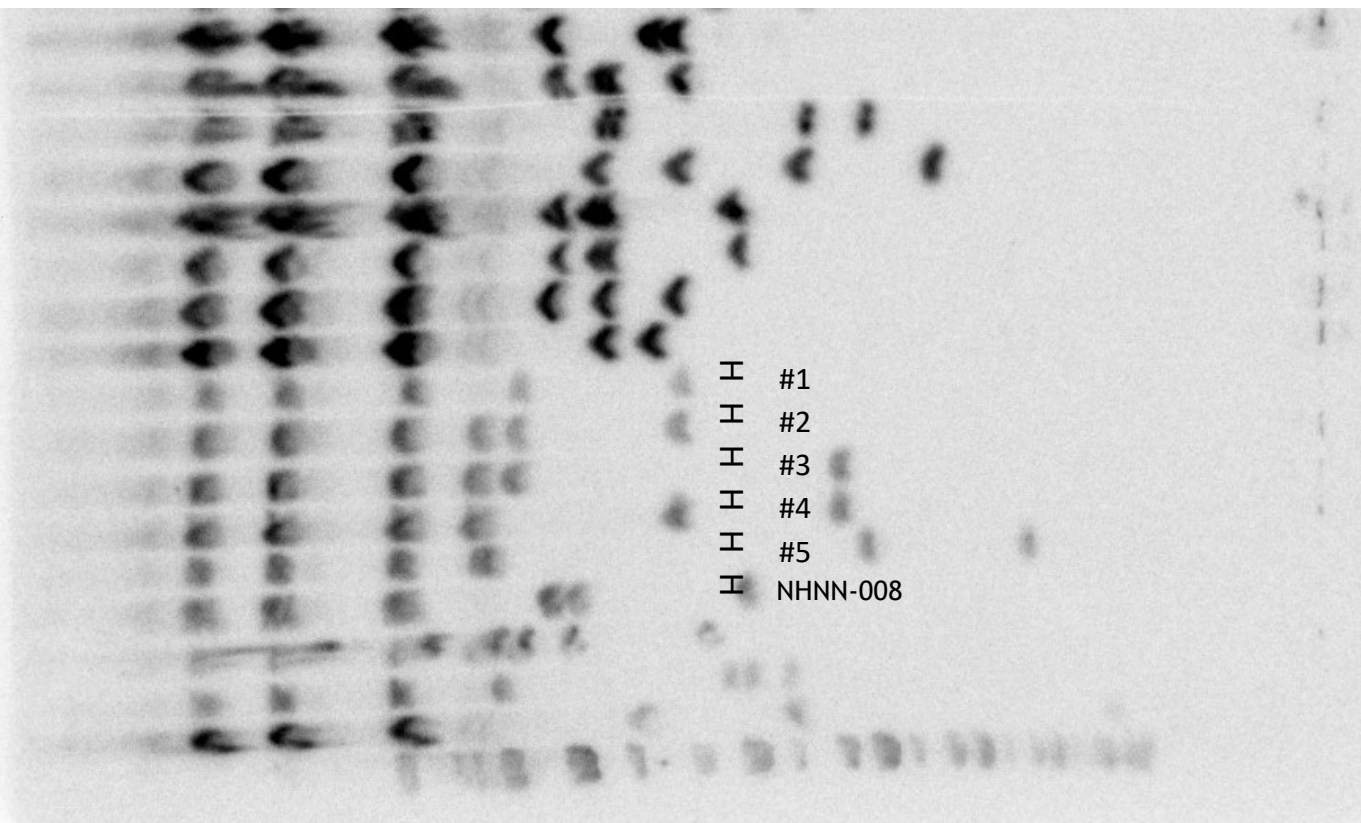

3) A

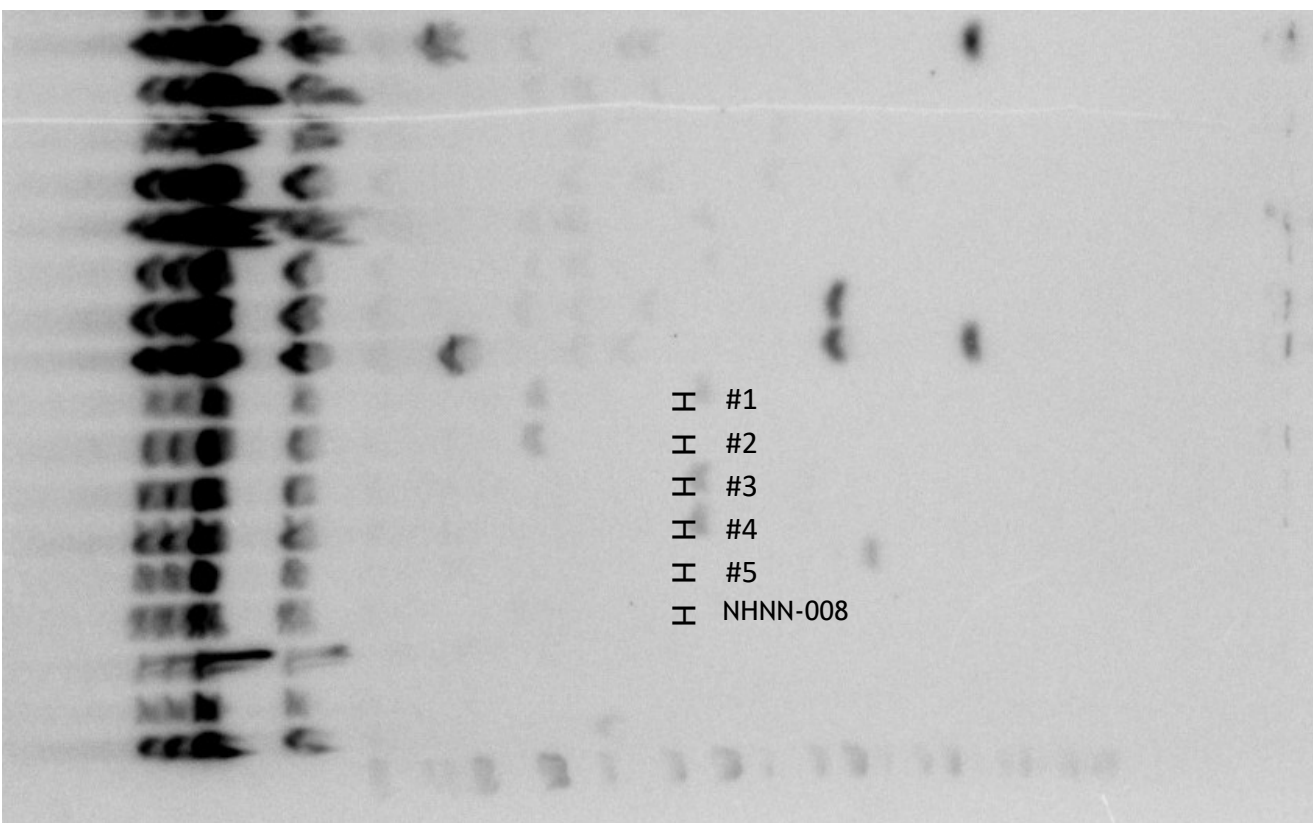

4) B

Figure S1: Original blot images.

Supplement: Supplementary file 1 [file biomolecules-13-01567-s001.zip › biomolecules-2583332-supplementary.pdf]
